# Supplementary material for: Efficacy Studies of a Trivalent Vaccine Containing PCV-2a, PCV-2b Genotypes and Mycoplasma hyopneumoniae When Administered at 3 Days of Age and 3 Weeks Later against Porcine Circovirus 2 (PCV-2) Infection
Source: Vaccines (Basel). 2022 Aug 1;10(8):1234. doi: 10.3390/vaccines10081234 (PMC9414577; doi:10.3390/vaccines10081234)
Supplement: Supplementary file 1 [file vaccines-10-01234-s001.zip › vaccines-1804025-supplementary.pdf]

Supplementary Materials

Table S1. Number of animals enrolled per each action and timepoint performed in clinical studies.

Weeks of age of study animals	Action performed	Num. of animals			
		Field trial A		Field trial B	
		Vaccinated pigs	Non-vaccinated pigs	Vaccinated pigs	Non-vaccinated pigs
<1	Body weight**	399	400	337	360
	Blood sampling	47	50	43	48
7	Faecal swabs	42	44	30	31
	Blood sampling	42	44	30	31
11	Faecal swabs	43*	43	42	46
	Blood sampling	44	43	42	46
16	Body weight**	325	323	378	389
	Faecal swabs	44	41	40	42
	Blood sampling	44	41	40	42
20	Faecal swabs	42	39	41	37
	Blood sampling	42	39	41	37
24-27	Body weight**	395	404	417	404
	Faecal swabs	39	40	48	53
	Blood sampling	39	40	48	53

*One missing faecal swab

** The number of animals weighed was not the same at each timepoint due to deviations occurring during the study (death animals or animals not found at the weighing moment)

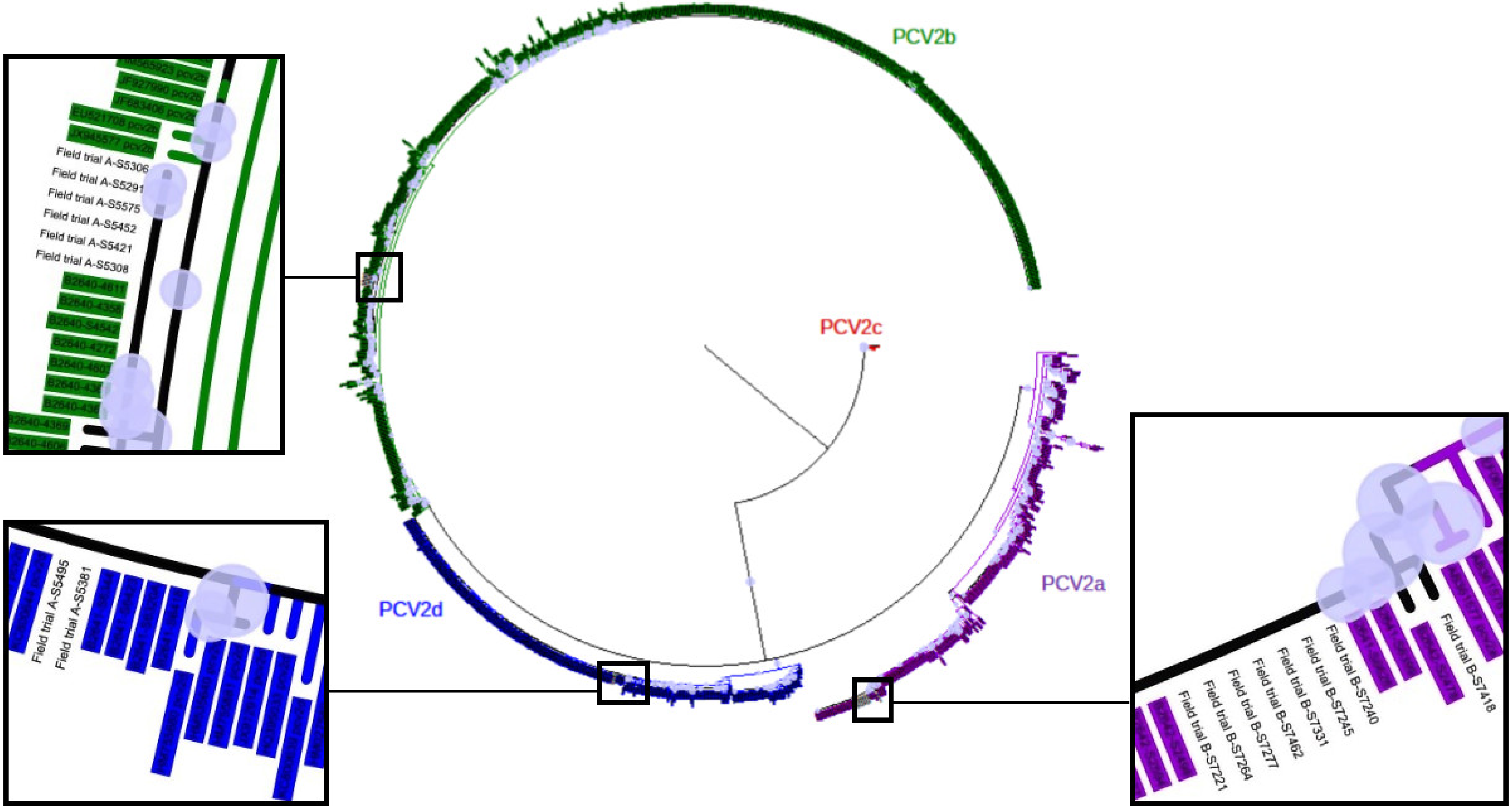

Figure S1: Phylogenetic tree derived from PCV-2 capsid protein (ORF2) sequences. Sequences from this study are indicated as Filed trial A or B plus the sample identification.
